# American Institute Farmers’ Club

**Published:** 1895-01

**Authors:** 


### AMERICAN INSTITUTE FARMERS' CLUB.

An important meeting of the American Institute Farmers' Club of New York City was held on Tuesday, January 8, 1895. The subject of tuberculosis in animals was one of the topics for discussion, and was participated in by Dr. Heath, of Brooklyn, who appealed for its solution in our herds and dairies on the basis of economy and justice to the public, to the farmer, and to the dairyman. He counseled against the reckless hand of slaughter, not because it was not swift and complete, but for the greater reason that the accumulation of property honestly is a slow and laborious process.

Veterinarian Moadinger, of Brooklyn, advocated preventive measures, the removal of tubercular animals from all herds, and said that this could be accomplished largely through better stabling, which would keep the animals from too close contact; good drainage, good food, pure water, and proper ventilation. He advocated greater care in the selection of attendants and milkers; that they be free from tuberculosis; that all fresh animals should be tested for admission to the herd.

Veterinarian Ackerman recommended the removal of all diseased animals from the herds; separation of milkers from breeding stock; spaying the milkers, and thus avoid the twofold drain of lactation and gestation which weakens the system and makes it a readier field for disease.

Veterinarian Finley in his remarks as to the tendency to recover, said there is a certain amount of resisting power in some, greater than in others, and greater at some times than at others.

Mr. E. G. Fowler, of the *American Agriculturist*, criticised strongly the movement in Massachusetts of general slaughter, and said that State was in a ferment from end to end. He condemned tuberculin as empirical, but when pressed for his reasons and for evidence of its unscientific value, he was compelled to acknowledged that this was only his personal opinion.

Veterinarian Pearson, of Philadelphia, strongly urged the value of tuberculin as a diagnostic agent, and completely combated the opinions expressed by Mr. Fowler with statistics, experience of many others, and an array of evidence that was too strong to be doubted.

Many farmers and dairymen spoke on the subject, covering the points of inbreeding, contamination by man, extermination of the diseased, and the quarantining of cases of human tuberculosis.

